# Spontaneous theory of mind in autism: are anticipatory gaze and reaction time biases consistent?

**DOI:** 10.3389/fpsyt.2024.1189777

**Published:** 2024-12-09

**Authors:** Keigo Onda, Rizal Ichwansyah, Keisuke Kawasaki, Jun Egawa, Toshiyuki Someya, Isao Hasegawa

**Affiliations:** ^1^ Department of Psychiatry, Niigata University School of Medical and Dental Sciences, Niigata, Japan; ^2^ Department of Physiology, Niigata University School of Medical and Dental Sciences, Niigata, Japan

**Keywords:** autism, theory of mind, spontaneous mentalizing, false belief test, anticipatory gaze, reaction time

## Abstract

**Background:**

Individuals with autism spectrum disorder (ASD) exhibit persistent deficits in social interaction and communication in adulthood. Pioneering studies have suggested that these difficulties arise from a lack of immediate, spontaneous mentalizing (i.e., theory of mind: ToM), specifically, an ability to attribute false beliefs to others, which should be usually acquired during neurotypical development. However, this view has been challenged by recent reports of nonreplications of spontaneous mentalizing, even in neurotypical adults.

**Objectives:**

We aimed to evaluate (1) whether measurements of spontaneous ToM in two representative paradigms, gaze bias in the anticipatory looking (AL) test and reaction time bias in the object detection (OD) test, are correlated in neurotypical adults and (2) whether these two measurements are altered in individuals with ASD.

**Methods:**

We developed a novel hybridized spontaneous false belief test combining the AL and OD paradigms to enable within-subject comparison of different spontaneous ToM measurements.

**Results:**

The results obtained with our hybridized test replicated the earlier positive evidence for spontaneous ToM in both AL and OD paradigms. Our results also revealed a correlation between the participants’ spontaneous gaze bias in the AL paradigm and reaction time bias in the OD paradigm, indicating that the participants who had spontaneously anticipated other’s false belief driven actions more quickly detected the object. We further found that spontaneous gaze and reaction time biases were altered in individuals with ASD. Finally, we ascertained those inclusions of these biases as diagnostic variables in a regression model improved the accuracy of diagnosing ASD. ASD diagnosis was best predicted by the model when variables obtained from both AL and OD methods were included in the model.

**Conclusions/implications:**

Our hybridized paradigm not only replicated spontaneous gaze bias in early AL studies and reaction time bias in the OD paradigms, but indicated significant correlation between them, suggesting that different implicit tasks tap the same spontaneous ToM in neurotypical adults. Group differences of these indices between ASD and neurotypical adult groups indicated that our task could help diagnose ASD, which is essential for evaluating the social difficulties that individuals with ASD face in adulthood.

## 1 Introduction

Autism spectrum disorder (ASD), a neurodevelopmental disorder with social and communication deficits as the core symptoms (1), is best explained as impairments of theory of mind (ToM) (2). ToM is defined as the ability to attribute mental states, such as beliefs or motivations, to other individuals (3). The normal acquisition and impairments of ToM are conventionally evaluated with verbal false belief (FB) tasks, such as the Sally and Ann task (4), which examines the ability to report that others have beliefs that are different from one’s own. The ages at which half of children pass these tasks are approximately 4 years and 9 years for children with neurotypical development and ASD, respectively; this difference is expected to discriminate between children with neurotypical development and those with ASD (5).

However, verbal (explicit) FB tasks might not be ideal for diagnosing ASD, as they require executive functions beyond ToM, such as linguistic ability and inhibitory control. Accumulating studies have emphasized that the ability to attribute FBs to others spontaneously and promptly is crucial for initiating and responding to social communication, as well as for building fluent social interactions. Explicit FB tasks may not effectively assess this ability. Even if adults with ASD pass explicit FB tests, they often continue to struggle with interpersonal communication and interactions, which are typically challenging for them (1, 6). Since the 2000s, Onishi and Baillargeon (7) Southgate et al. (8) and Kovács et al. (9) have analyzed the performance of young children who have not yet acquired language skills in nonverbal (implicit) ToM tasks. Implicit tasks measure spontaneity or promptness in FB attribution and should therefore be promising for the diagnosis of ASD. Senju et al. (10) analyzed the performance of adults in the anticipatory looking (AL) task, which is one of the major implicit tasks used to measure theory of mind implicitly by tracking spontaneous eye movement patterns while watching a FB task movie. They reported differences between those with neurotypical development and those with ASD, hypothesizing that “the absence of spontaneous theory of mind would cause difficulty in social interaction and communication, even in adults with high verbal and cognitive skills” (11).

However, many recent attempts to replicate earlier findings (12–19) using implicit FB tasks have failed, raising serious concerns about whether implicit tasks truly measure ToM. The disadvantages of these tasks include a very high dropout rate and difficulty in participant engagement following implicit task instructions (17). Most recent nonreplicated studies using the AL paradigm developed by Southgate et al. (8) reported high dropout rates of over 40% (12), and this method is difficult to replicate even in neurotypical adults and children (14, 15). Kulke and Hinrichs (20) attempted replication in adults by constructing a realistic interactive paradigm (realistic paradigm) with a storyline in the task movie, but there was no significant improvement in dropout rates or test results compared with those of the original study. In contrast, the object detection (OD) test (9), a test used to evaluate the degree of implicit FB attribution to others by measuring reaction time, is more engageable owing to the task achievement goal of button pressing. The irrelevance between the task instructions and the agent’s belief is expected to reduce dropout rate and learning effects. Therefore, we thought that a combination of these two methods could provide a more reliable testing approach, as the OD requires a certain level of active participation. In addition, we developed several improvements to this task. In general, nonverbal (implicit) FB tasks are susceptible to context-specific variables in the experimental environment and the content of the movies (21), which may be one of the reasons why consistent results have not been obtained within and across tasks. In the present study, we took several steps to reduce the influence of external factors as much as possible. We used a minimally stimulating experimental booth and non-invasive head fixation to obtain eye-movement measurements, thereby directing attention to the monitor. The total duration of the experiment was shortened to approximately 20 minutes, given the limitations of participant concentration. The contents of the movies composed a storyline, and the viewpoint was from the agent’s perspective, in accordance with the OD. To increase the sensitivity of the eye-movement measurement, a sufficient visual field range with a maximum visual field angle of 8° was secured. Furthermore, by combining the AL and OD paradigms, the task instructions were limited to the minimum necessary, such as “Please pull the lever on the side where the object reappeared at the end of the movie”, which were irrelevant to the explicit comprehension of the agent’s intension in the AL paradigm. In the present study, the task movies were presented repeatedly; however, we prevented learning effects by interrupting the movie at the object detection scene instead of showing the last scene as in the AL paradigm (the scene in which the agent captures the object). No significant learning effect was present in the pilot or present study (**Supplementary Figures 2**, **6**).

We developed a paradigm that measures AL eye movements during the OD task in adult participants to identify differences in spontaneous FB attribution abilities (i.e., ToM) between neurotypical adults and adults with ASD. We initially used this paradigm to examine our first research question: Is spontaneous ToM reproducible in neurotypical adults? Specifically, we tested whether there is a significant gaze bias indicating the implicit attribution of FBs to agents in movies among neurotypical adults (Hypothesis 1: H1). Since the reproducibility of the AL paradigm should depend on whether participants can anticipate the agent’s behavior, the first step was to determine whether spontaneous gaze of neurotypical adults reliably anticipated the agent’s behavior in the true belief (TB) condition in our paradigm. Once this prerequisite was established, we then asked whether anticipatory gaze behavior was confirmed under FB conditions. Reproducibility of the anticipatory gaze bias in the FB condition would support the claim of the existence of spontaneous ToM.

Next, while explicit ToM has been consistently observed across various tasks, no systematic correlations have been found between the different types of implicit tasks (22, 23). This leads to the second research question: Is there a concept of spontaneous ToM that is tapped into by different tasks? In other words, is gaze-based implicit ToM defined with the AL paradigm equivalent to reaction time-based spontaneous ToM defined with the OD? Our newly constructed FB task provides results for each paradigm simultaneously, allowing direct comparison of both paradigms. Using this task, we tested the following hypotheses that anticipatory gaze behavior and reaction-time bias on the object detection test are consistent within individuals and that anticipatory gaze behavior and reaction-time bias on the object detection test are correlated, with anticipatory gaze affecting reaction time (H2). The correlation of these two independent measures would support the existence of task-invariant spontaneous ToM.

The present study aims to resolve the long-standing controversy over whether the AL paradigm truly measures spontaneous ToM, which has been contentious for year. In the pilot study, the AL and OD results were significantly correlated (**Supplementary Figure 4**; the number of neurotypical adults participating in the study was increased to 20 for statistical evaluation following registration). Furthermore, if spontaneous ToM is reproducible across tasks among neurotypical adults, it will lead to a third research question: does spontaneous ToM differ between neurotypical adults and ASD adults (H3)? If ASD adults demonstrate impairments in spontaneous FB formation, then the proportion of correct first looks and the differential looking time score (DLTS) measured by the AL method and the ToM index value (see below) in the OD of ASD adults are expected to be significantly lower than those of neurotypical adults.

Finally, we examined the relationship between each measurements and the autism-spectrum quotient (AQ) score or ASD diagnosis. The pilot study indicated that the AQ score was correlated with the ToM index, suggesting that individuals with higher AQ scores may exhibit differences in gaze behavior and/or reaction times (H4). Furthermore, we tested how these measurements affect the diagnosis of ASD by comparing them in a regression model with the first look ratio or differential looking time score (obtained from the AL task) and reaction time (obtained from the OD task). In other words, we tested a hypothesis that the best model for explaining the diagnosis with or without ASD included both the AL measurement and the ToM index rather than the ToM index alone (H5).

We used this modified implicit FB task to overcome methodological problems and evaluate the reproducibility of the FB task and the differences in implicit ToM in adults with ASD. This study is expected to provide important insights for future ASD diagnostics and contribute to the understanding of social behavior.

## 2 Materials and methods

### 2.1 Ethics information

This study was conducted in accordance with the Declaration of Helsinki and approved by the Ethics Committee of Niigata University (approval number: 2021-0333).

### 2.2 Design

The experimental procedures were as follows. A participant faced the monitor, rested his or her head on a noninvasive head fixation frame (constructed in-house), and performed the task in an experimental booth with minimal external stimuli. The experiment took approximately 20 minutes and was designed to be as simple as possible to allow it to be performed during outpatient clinic hours.

**Figure 1:**
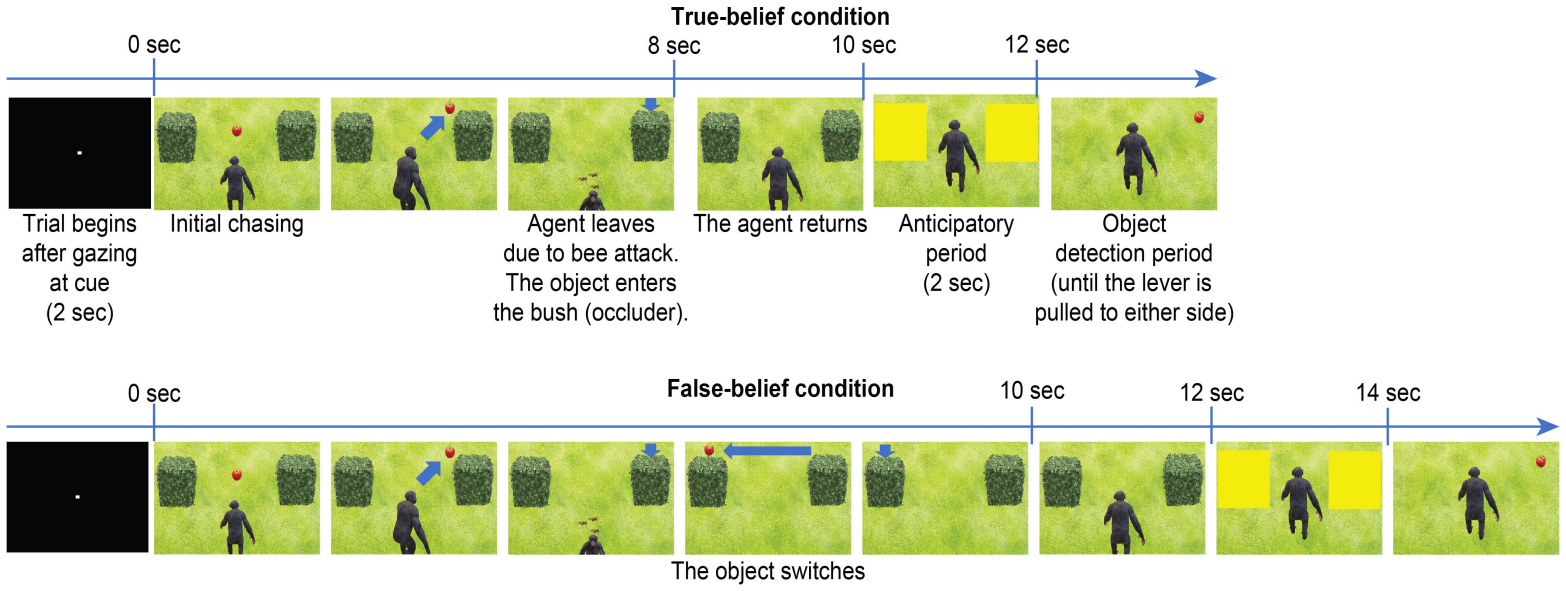
Events and timing of the trials of the TB (P+A+) and FB (P−A+) conditions. The upper figures depict the familiarization and test trials of the TB condition (P+A+). The lower figures depict the test trial of the FB condition (P−A+), where the object switches sides, reappearing on the unexpected side. The AOIs are depicted as yellow rectangles after the agent returns. The direction the agent turns is counterbalanced across trials.

The participants were required to maintain their gaze within a visual angle of 1–3°centred on a white spot (0.3°) on a 22-inch LCD monitor (BenQ, XL 2411 T, Taipei, Taiwan) with a refresh rate of 100 Hz and a viewing angle of 30°×20°from 50 cm away. Gaze positions were noninvasively captured and calibrated with an infrared camera system at a sampling rate of 300 Hz (irec_2HS, https://staff.aist.go.jp/k.matsuda/eye/). For calibration, participants were required to successively maintain their gaze on 9 spots—4 in the 4 corners of a square display area on the monitor, 4 in the midpoints of each side of the square, and 1 in the center of the square. Task control and data acquisition were performed with custom-made software (NSCS, Niigata, Japan) and PCI extensions for instrumentation (PXI) running on a real-time LabVIEW system (National Instruments, TX, USA). Movies were presented with visual presentation software (Active STIM, http://www.danko-nikolic.com/activestim/) and synchronized with the PXI system. Gaze data were sampled at 1 kHz using this system. We prepared animated movies in which an agent tracked an object (**Figure 1**) edited with PowerPoint^®^ (Microsoft Corp., WA, USA). The sizes of the left and right areas of interest (AOIs) during the eye-movement measurement period were 267×202 pixels (84 dpi).

#### 2.2.1 Combination of the AL and OD paradigms

Exploiting the designs of the AL and OD paradigms, namely, that the AL paradigm measures anticipatory eye-movement data before the reappearance of an object (pre-evaluation) and that the OD paradigm measures the reaction time after the reappearance of the object (post-evaluation), we developed a hybrid paradigm combining the two (see the **Supplementary Movie**). The first half of the movie, which took 10 or 12 seconds, followed the AL paradigm, and the second half of the movie naturally led to the OD paradigm. At the end of the AL paradigm, the AOIs covering the left and right occluders (in this case, bushes) turned yellow for 2 seconds, during which the directions of the first look and DLTS were measured. In the second half of the movie (OD paradigm), the yellow windows and the occluders successively disappeared, which revealed the object hidden in one of the two occluders. The reaction time was defined as the latency from the object reappearance until the participants’ response (pulling the lever in the left or right direction). The trial ended when the lever was pulled or when the 2-second time limit expired. In this task, the participants were just instructed to “please pull the lever in the direction of the object when it reappears”, similar to the conventional instructions of the OD task. Consequently, the participants completed the entire task while they were unaware of being tested in the FB task, which satisfied the definition of an implicit FB task (i.e., the intent of the task was not indicated verbally). In addition, since the FB AOI and the control AOI were measured on the same screen, differences in spatial attention due to the FB were directly compared between the left and right sides of the same screen.

**Supplementary Figure 1** presents an example of a FB formation scenario. The agent tracks and sees the object until the object is first hidden; then, the agent turns around. When the agent is absent, the object moves to the opposite side and disappears from view, by which the participants can attribute FBs to the agent. The agent then returns and attempts to find it by searching the object’s original position on the basis of the agent’s FB. In this case, the first half of the movie (AL paradigm) is a FB condition, whereas the second half of the movie (OD paradigm) is a P−A+ condition because the location where the object reappears is as expected for the agent (A) but unexpected for the participant (P). Therefore, the participant’s lever-pull response in the P−A+ condition should be slower than that in the P+A+ condition or TB condition, in which the object reappears where both the participant’s and the agent’s expectations are met. However, if the participant’s spatial attention is also directed to the agent’s FB to some extent, then the response in the P−A+ condition would be faster than that in the P−A− condition, in which the object reappears where both the participant and the agent unexpected.

The order of all of the movies was randomized and counterbalanced according to the object position; thus, all of the participants viewed a total of 48 movies [3 (agent−object variation) × 4 (condition: P+A+, P−A−, P−A+, and P+A−) × 2 (side: left and right) × 2 (direction in which the agent rotates: left and right)]. The participants were provided only the initial instructions for the OD task. Therefore, participants were blinded to the aim and subject of the study. The participants were also blinded to the order of the conditions to which they were assigned.

### 2.3 Sampling plan

Individuals with ASD were recruited from the outpatient psychiatric department of Niigata University Medical and Dental Hospital and were adults with no language or intellectual disabilities. The control participants were recruited via social media as well as paper announcements. They consisted of age- and IQ-matched participants (aged 20 years or older) without a history of neurological or psychiatric disorders. IQ scores were assessed with a seven-subtest short form of the Wechsler Adult Intelligence Scale (24, 25), except when participants had already completed a full WAIS-IV test. Sample size estimates were calculated via G*Power (26). The planned statistical analysis involved repeated-measures ANOVAs: 1) For the AL paradigm, the repeated-measures ANOVA had the following design: 2 (group: ASD vs. neurotypical (NT)) × 2 (condition: TB or FB); 2) For the OD task, the repeated-measures ANOVA had the following design: 2 (group: ASD vs. NT) × 4 (condition: P+A+, P−A−, P−A+, or P−A−). The alpha error, power, and effect size were set to 0.05, 0.95, and moderate, respectively. The total number of participants required was calculated as 36–50. In anticipation of a certain number of participants meeting the inclusion criteria, 20 participants were included in each group. The inclusion criteria were as follows: 1) those who completed familiarization (defined below), 2) subjects with a correct response rate in the OD task above 90%, and 3) control individuals with an autism-spectrum quotient (AQ) (27) score of 31 or lower. With respect to the first criterion, four TB-condition videos were presented in familiarization trials prior to the test trial, and those who exhibited at least one correct look in the last two (of four) trials (hereafter, those who completed familiarization) were included. The subjects provided written informed consent before taking part in the study.

### 2.4 Analysis plan

The three main steps of the present analysis as follows: (1) performing a mixed-model repeated-measures ANOVA for each measurement obtained in the FB task for adults with ASD and neurotypical adults and determining whether group differences occur in each task; (2) performing a correlational analysis between each measurement of the two belief tasks to determine if the ALT and OD paradigms are consistent; and (3) examining which variables should be included in the best-fit model for ASD diagnosis.

#### 2.4.1 Mixed-model repeated-measures ANOVA for each measure

The main measurements included the proportion of correct first looks and the DLTS in the AL paradigm, where the first look was defined as the first time the participant’s gaze remained within either the left or right AOI for more than 100 ms during the analysis period. The DLTS was defined as the ratio of the difference between the target and nontarget looking times to the total looking time.


DLTS = (target looking time-nontarget looking time)/(target looking time + nontarget looking time)

However, this formula tends to be biased towards 1 or −1 regardless of the length of gazing time if, for example, the target or nontarget looks are fleeting. For this reason, the denominator was fixed at 2,000 ms for the eye-movement measurement period, and the following modifications were made to transform the DLTS data into a parametric distribution:


DLTS = (target looking time-nontarget looking time)/2000

The other measure was the reaction time on the OD task, which was defined as the time from the object’s reappearance to the time when the lever was pulled.

ANOVAs were used to investigate the effects of location (expected or unexpected) or reaction time, belief condition (TB or FB condition for the AL paradigm; P+A+, P−A−, P−A+, or P+A− for the OD paradigm) and the interaction of both factors. Bayes factor (BF) with an evidence threshold of 3 was determined using the proportion function of the BayesFactor package in R (28).

#### 2.4.2 Correlation analysis between the DLTS (AL measurement) and the reaction time (OD measurement)

A correlation analysis was conducted using trial-by-trial values of the DLTS and reaction time to examine the relationship between the AL and OD measurements. The reaction time was standardized for each subject. We used the z score function in MATLAB to return a z score such that the within-subject mean was centered to 0 and scaled to a standard deviation of 1.

#### 2.4.3 Regression analysis and model selection

In the present experiment, the dependent variable was the AQ score (1), but in the main experiment, logistic regression analysis was conducted using variable 2, i.e., the NT or ASD group, and the independent variables for each measure.

1. Dependent variable p_i_: Participant (i) AQ score
2. Dependent variable p_i_: Participant(i) group, NT (0) or ASD (1)

Independent variable xi: Reaction time (ToM index), DLTS (FB), or AQ score

1. p_i_ = β_0_ + β_1_x_i1_ + β_2_x_i2_ + β_3_x_i3_
2. log (pi/1 - pi) = β_0_ + β_1_x_i1_ + β_2_x_i2_ + β_3_x_i3_

As a supplementary measure, the AQ was used to assess the ASD severity, and the DSM-5 criteria were used to diagnose ASD.

## 3 Results

### 3.1 Testing implicit false belief attribution for neurotypical and ASD adults

Twenty participants aged 20 years or older were included in the neurotypical adult group (10 of whom were female, aged 26.7 ± 5.8 years). Five participants were excluded, 4 of whom failed the familiarization trial or poor eye measurements and one due to an AQ score > 32 (exclusion rate of 20%). Twenty participants with ASD were included in the ASD group (9 of whom were female, aged 31.6 ± 9.4 years). Six ASD participants were excluded: 5 due to failure in the familiarization trial or poor eye measurements and one with comorbid mild intellectual disability (exclusion rate of 23%). The order in which the movies were presented, the direction in which the agent turned away, and the position of the object’s appearance were counterbalanced. Following object reappearance, all of the participants pulled the lever in the correct direction with an accuracy of over 95%. As a supplementary psychological test, the AQ (mean score, 17.7 ± 5.5 for NT, 33.8 ± 5.2 for ASD [Welch’s t-test, p < 0.001]) and the WAIS-IV (mean score, 103.9 ± 10.3 for NT, 98.8 ± 15.6 for ASD [Welch’s t-test, p = 0.295]) were used.

A hybrid paradigm combining both AL and the OD paradigms (see the Methods for details) was designed to determine whether neurotypical adults exhibit significant gaze bias and reaction-time bias, indicating the implicit attribution of FBs to agents in movies and whether these two successively acquired measures are consistent within individuals. In the last scene, the movie is terminated when the participant pulls a lever to the left or right after the object reappears. The movie does not show the agents pursuing their prey to the end to minimize the learning effect. This methodology followed previous work (29), in which the interruption of FB-based searching for the object prevented the modulation of gaze behavior (30). To assess this learning effect, we first divided all of the data into four time bins to examine the learning effect across trials within subjects in the neurotypical group (**Supplementary Figures 6A–C**). Two-way ANOVA indicated that there were no significant main effects of the condition (TB or FB condition) [Bayes factor (BF) = 1.464 for the first correct look ratio, BF = 0.267 for the DLTS], time bin [BF = 0.168, BF = 0.173, respectively] and no interaction between the condition and time bin [BF = 0.311, BF = 0.300, respectively]. *Post hoc* multiple comparisons via two-tailed tests were not significant [Holm test, BF < 3] for both time-bin combinations. Regarding reaction time (OD measurement), a two-way ANOVA indicated that there was no significant main effect of time bin [BF = 0.465] or a significant interaction between the condition and time bin and condition [BF = 0.034]. *Post hoc* multiple comparisons via two-tailed tests were not significant [Holm test, BF < 3] for both time-bin combinations. These results indicated that there was no learning effect for any measurements (the first look ratio, DLTS, or reaction time). Therefore, in this main experiment, the data for analysis were summarized as trial averages for each condition. However, to analyze the correlation between the DLTS and reaction time, data for each trial were used.

**Figure 2:**
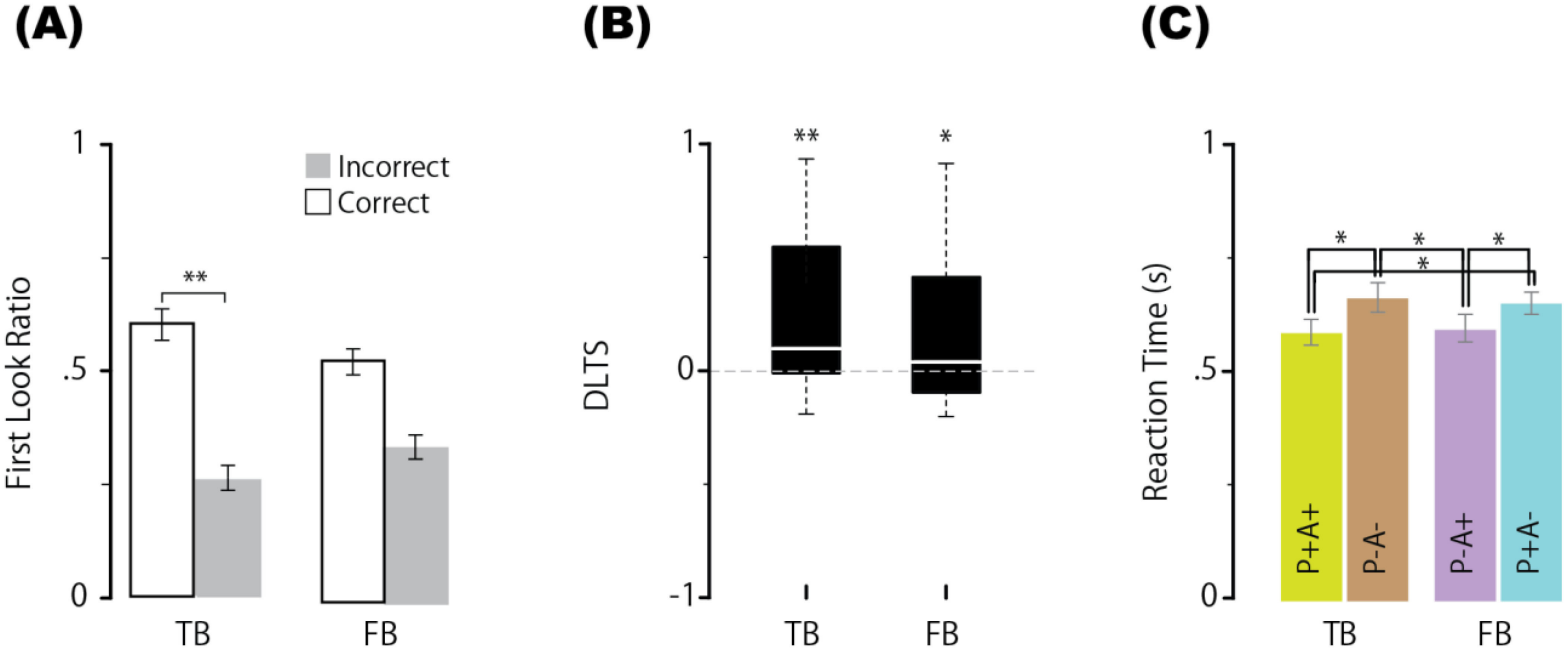
Proportion of first looks (correct or incorrect) for each condition **(A)**, differential looking time score (DLTS) **(B)**, and reaction times in the P+A+, P−A−, P−A+, and P+A− conditions **(C)** in neurotypical group. Error bars represent the standard error of within-subject effects. ** indicates a BF > 10, and * indicates a BF > 3. N=20. TB, true belief; FB, false belief.

In the neurotypical group, regarding the proportion of correct first looks in the main experiment (**Figure 2A**), a Bayes factor analysis of the two-way ANOVA showed that the BF value for the main effect of condition (TB or FB condition) was negligible [BF = 0.224, F(1,19) = 0.51, p = 0.483, ηp² = 0.026], the BF value for the main effect of correct/incorrect first looks was very strong [BF = 37804, F(1,19) = 10.664, p = 0.004, ηp² = 0.36] and the BF value for the interaction was negligible [BF = 0.903, F(1,19) = 3.671, p = 0.07, ηp² = 0.162]. These findings suggest that the ratio of correct first looks significantly differed from that of incorrect first looks in any condition. For reference, we assumed a simple main effect in the interaction and performed multiple comparisons (two-tailed test) with paired means for each level. The BF value for the main effect of the correct/incorrect first look-in-the TB condition was significant [BF= 76.435, F(19,19) = 8.891, adjusted p = 0.01, ηp² = 0.319] but not significant in the FB condition [BF= 1.015, F(19,19) = 2.678, adjusted p = 0.118, ηp² = 0.124]. Specifically, the mean correct first look ratio in the TB condition (0.616) was significantly greater than the mean incorrect first look ratio (0.261) but not in the FB condition (0.529 vs. 0.334). Next, a BF analysis with one-way ANOVA for the DLTS showed that the BF value for the main effect of the mean DLTS significantly differed from the chance level of 0 in the TB condition [mean = 0.268, BF = 42.763, F(1,19) = 11.134, p = 0.003, ηp²= 0.369], and even in the FB condition [mean = 0.188, BF = 3.738, F(1,19) = 5.281, p = 0.033, ηp² = 0.217] (**Figure 2B**). Third, regarding the reaction time, a BF analysis of the one-way ANOVA was performed across conditions (P+A+, P−A−, P−A+, P+A−), and the main effect of condition was highly significant [BF = 486.562, F(3,57) = 9.26, p < 0.001, ηp²= 0.328]. Multiple comparisons (two-tailed, Holm’s method) using paired means for each level revealed that the mean reaction time in the P+A+ condition (580 ms) was significantly shorter than that in the P−A− condition (655 ms) [BF = 19.087, t(19) = 3.5538, adjusted p = 0.0051], and the mean reaction time in the P−A− condition was significantly longer than that in the P−A+ condition (587 ms) [BF = 12.806, t(19) = 3.348, adjusted p = 0.0060]. These results replicate those of previous studies, such as Kovács et al. (9), indicating that in addition to the participants’ own beliefs, the agent’s beliefs influenced the reaction time. Additionally, the mean reaction time in the P+A− condition (643 ms) was significantly longer than that in the P+A+ condition (579 ms) [BF = 20.581, t(19) = 3.5923, adjusted p = 0.0073] and P−A+ condition (587 ms) [BF = 12.806, t(19) = 2.9, adjusted p = 0.013] (**Figure 2C**). Therefore, simply seeing the agent automatically made neurotypical participants attribute beliefs to the agent and that the agent’s beliefs might be represented and sustained like the participants’ own beliefs.

**Figure 3:**
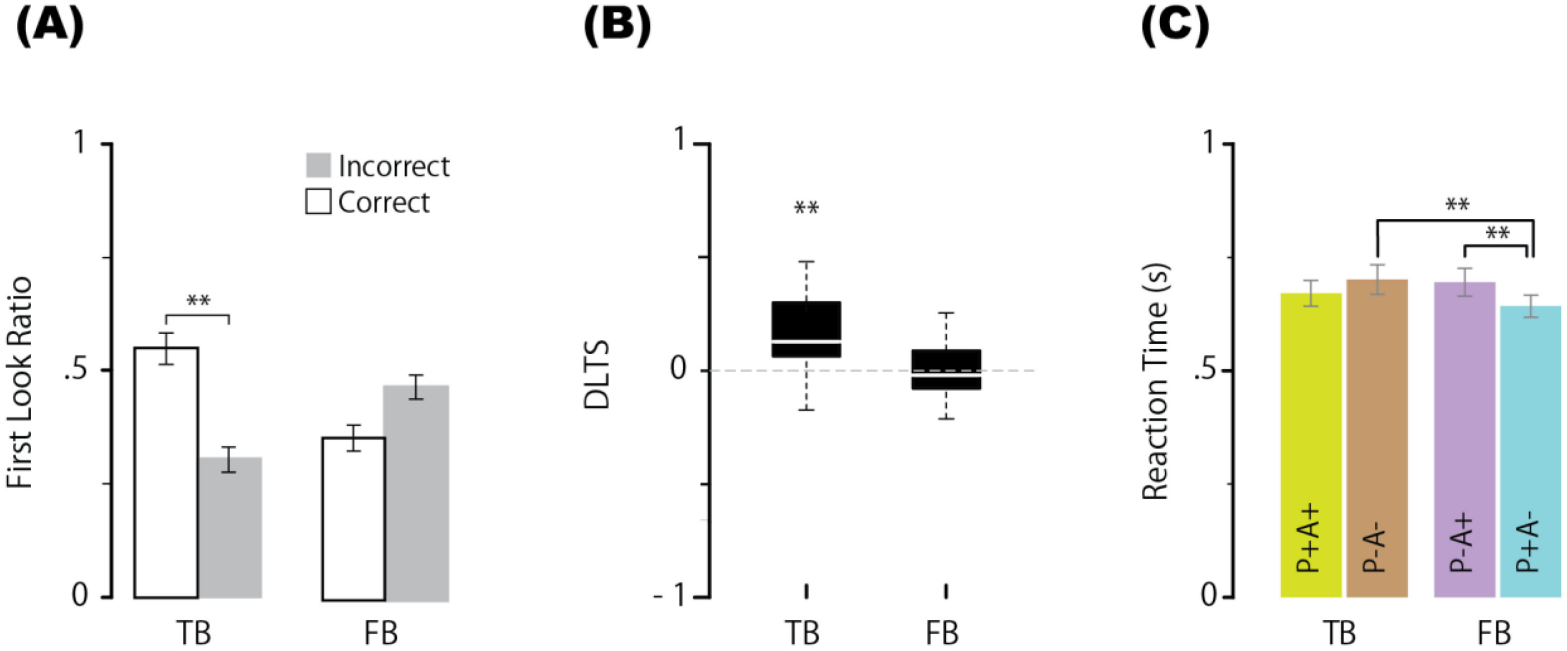
Proportion of first looks (correct or incorrect) for each condition **(A)**, DLTS **(B)**, and reaction times in the P+A+, P−A−, P−A+, and P+A− conditions **(C)** in ASD group. Error bars represent the standard error of within-subject effects. ** indicates a BF > 10. N=20. TB, true belief; FB, false belief.

In sharp contrast to the results for the neurotypical group, a two-way ANOVA for the first look ratios in the ASD group revealed that the main effects of neither condition [BF = 0.229, F(1,19) = 0.024, p = 0.879, ηp² = 0.001] nor correct/incorrect first looks [BF = 0.82, F(1,19) = 2.092, p = 0.164, ηp² = 0.099] were significant. The BF value for the interaction was very strong [BF = 919173, F(1,19) = 31.744, p < 0.001, ηp² = 0.626]. One-way ANOVA revealed that the BF value for the main effect of the correct/incorrect first look ratio was significant in the TB condition [BF = 117.790, F(19,19) = 16.907, adjusted p < 0.001, ηp² = 0.471] but nonsignificant in the FB condition [BF = 2.202, F(19,19) = 4.269, adjusted p = 0.052, ηp² = 0.184]. In the TB condition, the mean correct first look ratio (0.528) was substantially greater than the mean incorrect first look ratio (0.302) but not in the FB condition (0.357 vs. 0.470) (**Figure 3A**). Next, a BF analysis with one-way ANOVA for the DLTS showed that the BF value for the main effect of the mean DLTS in the TB condition significantly differed from the chance level [mean = 0.154, BF = 412.097, F(1,19) = 17.041, p < 0.001, ηp² = 0.473] but not in the FB condition [mean = −0.035, BF = 0.638, F(1,19) = 1.478, p = 0.238, ηp² = 0.072] (**Figure 3B**). Third, regarding the reaction time, a BF analysis of the one-way ANOVA was performed across conditions, and the main effect of condition was significant [BF = 45.706, F(3,57) = 6.634, p < 0.001, ηp²= 0.259]. Multiple comparisons using paired means for each level revealed that the mean reaction time in the P+A+ condition (669 ms) was not significantly shorter than that in the P−A− condition (700 ms) [BF = 0.681, t(19) = 1.59, adjusted p = 0.171], and the mean reaction time in the P−A+ condition (694 ms) was not significantly shorter than that in the P−A− condition [BF = 0.248, t(19) = 0.3847, adjusted p = 0.259]. The mean reaction time in the P+A− condition (641 ms) was significantly shorter than that in the P−A− condition [BF = 44.435, t(19) = 3.981, adjusted p = 0.002] and P−A+ condition [BF = 2259.467, t(19) = 5.963, adjusted p < 0.001] (**Figure 3C**). Overall, the results of the FB condition in the ASD group were reversed compared with those in the neurotypical group. For reference, multiple two-tailed comparisons were conducted for each measurement to evaluate the main effects between groups (neurotypical vs. ASD) under the FB condition. As a result, significant main effects were found across all measurements: for the correct first look ratio (0.529 vs. 0.357), BF = 5.093, F(1,38) = 6.678, adjusted p = 0.027, ηp² = 0.163; for DLTS (0.188 vs. −0.035), BF = 3.802, F(1,38) = 6.61, adjusted p = 0.028, ηp² = 0.148; and for reaction time under the P−A+ condition (587 vs. 694), BF = 10.139, F(1,38) = 9.385, adjusted p = 0.008, ηp² = 0.198.

**Figure 4:**
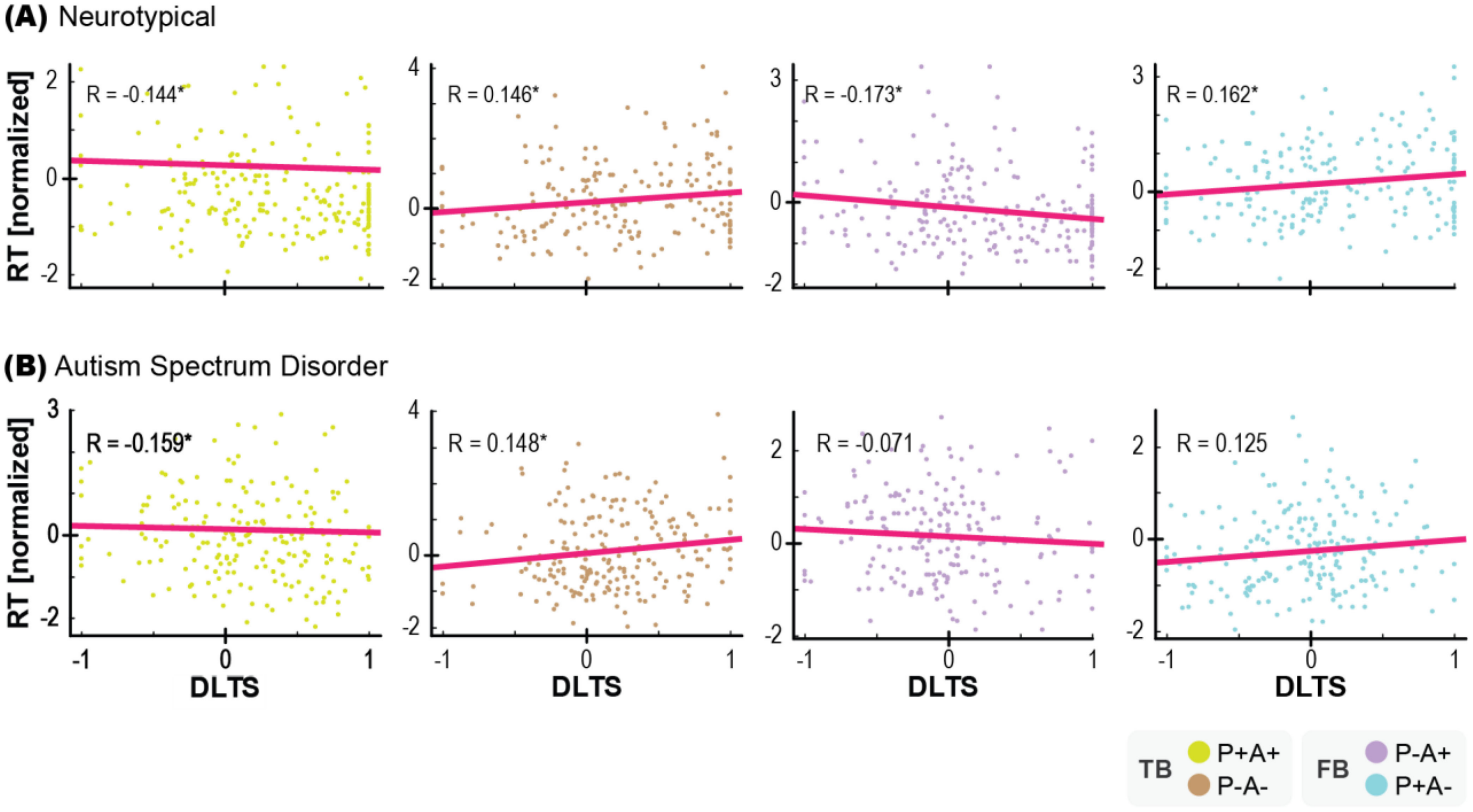
Relationship between the DLTS and reaction times in each trial in each condition in neurotypical group **(A)** and ASD group **(B)**. Reaction times were normalized for each subject. * indicates p < 0.05. N=20 each.

To further confirm the relationship between the AL and OD paradigms, a correlation analysis was conducted using the trial-by-trial DLTS and reaction time. The reaction time was normalized for each subject. The results in the neurotypical group revealed moderate to strong correlations between the DTLS and reaction time in the TB (P+A+) condition [r = −0.144, p = 0.034, BF = 2.76, ρ_95% CI: −0.267 to −0.143], TB (P−A−) condition [r = 0.146, p = 0.044, BF = 2.341, ρ_95% CI: 0.022 to 0.148], FB (P−A+) condition [r = −0.173, p = 0.011, BF = 7.11, ρ_95% CI: −0.296 to −0.168] and FB (P+A−) condition [r = 0.162, p = 0.021, BF = 4.286, ρ_95% CI: 0.036 to 0.162]. Thus, there was a significant correlation between the data obtained with the two paradigms, indicating that anticipatory gaze, reflecting belief attribution to the agent, strongly affected reaction time (**Figure 4A**). In the ASD group, the correlation was observed only in the TB conditions, i.e., the TB (P+A+) condition [r = −0.159, p = 0.025, BF = 3.703, ρ_95% CI: −0.286 to −0.156], TB (P−A−) condition [r = 0.148, p = 0.031, BF = 2.979, ρ_95% CI: 0.030 to 0.150], but not in the FB (P−A+) condition [*r* = −0.071, p = 0.322, BF = 0.444, *ρ*_95% CI: −0.207 to −0.083] or the FB (P+A−) condition [r = 0.125, p = 0.076, BF = 1.452, ρ_95% CI: 0.017 to 0.127] (**Figure 4B**). These findings provide evidence that AL and OD are different ways of assessing the same symptom of lack of spontaneous mental attribution to others, which is one of characteristic of ASD.

### 3.2 Regression analysis and model selection for AQ score and ASD diagnosis

In addition, to examine the contribution of each measurement (first look ratio, DLTS, or reaction time) to the AQ score, a regression analysis was conducted with the AQ score as the dependent variable and each measurement as the independent variable. A BF analysis was subsequently conducted for the following interaction models, and model selection was subsequently performed (**Supplementary Table 2**).

**Y: AQ (autism-spectrum quotient)**

**x1: ToM index (reaction time difference between the P−A−and P−A+ conditions)**

**x2: Proportion of correct first looks (TB condition)**

**x3: Proportion of correct first looks (FB condition)**

**x4: Differential looking time score (TB condition)**

**x5: Differential looking time score (FB condition)**

**Y ~ x1 + x2 + x3 + x4 + x5 (for regression analysis and model selection)**

In the regression analysis with AQ as the dependent variable, none of the regression equations were significantly correlated with AQ [BF < 3, p > 0.1]. The AQ score was not correlated with any measurements.

**Figure 5:**
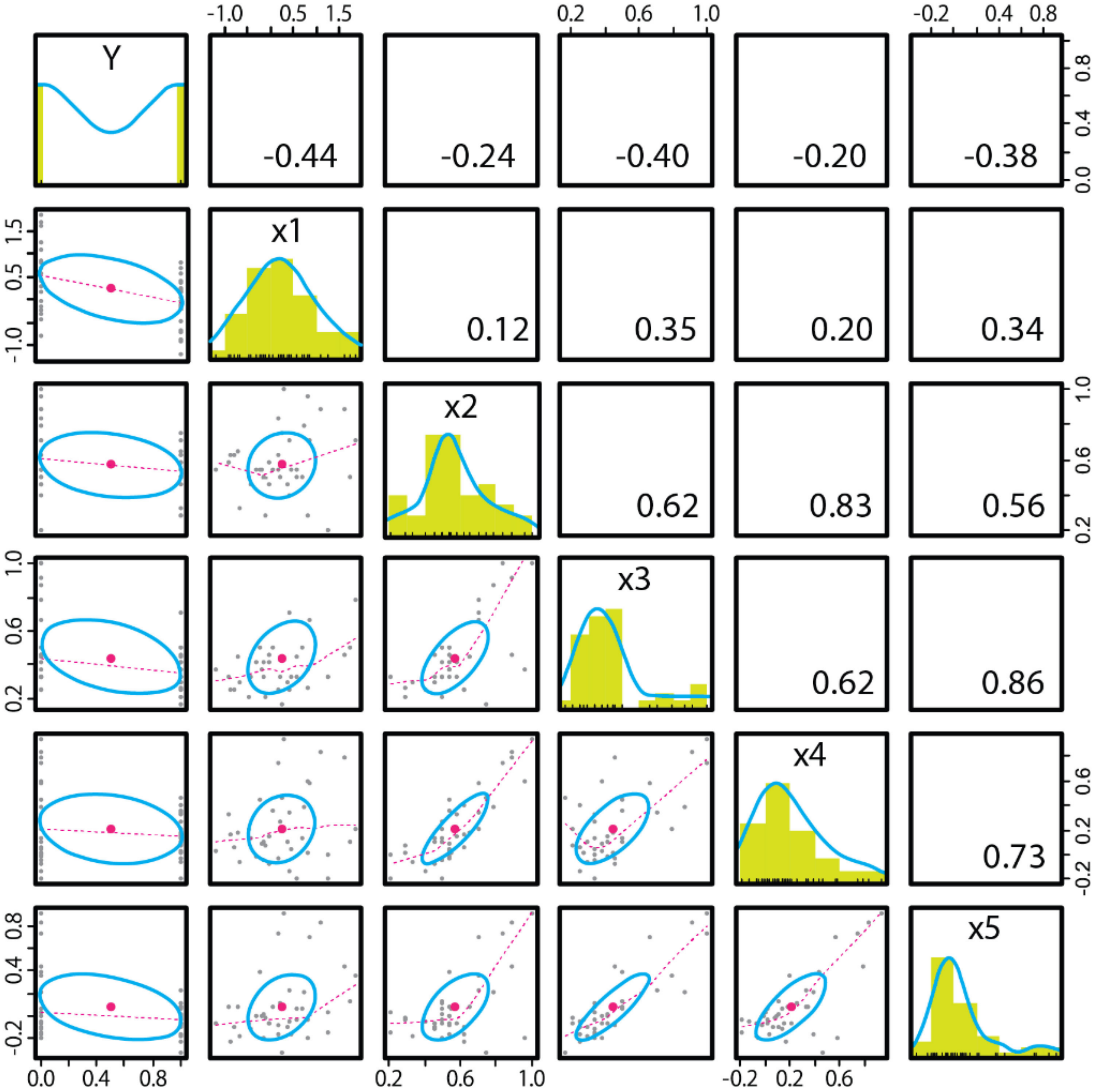
Correlation coefficient between the binary variable in ASD diagnosis (on the Y axis) and all measurements (on the X axis). N=40.

Next, we constructed a model equation with the diagnosis of ASD or neurotypical as the dependent variable (**Table 1**, **Figure 5**).

**Table 1:** Regression results and model selection with the ASD diagnosis (Y) as the dependent variable and the ToM index (x1), FL in the FB condition (x3) and DLTS in the FB condition (x5) as the independent variables. ToM_id, ToM index; DL_FB, DLTS in the FB condition; FL_FB, correct first look ratio in the FB condition; AIC, Akaike's Information Criterion; BIC, Bayesian information criterion.

	Model (n = 40)					
	Y~x1	Y~x3	Y~x5	Y~x1+x3	Y~x1+x5	Y~x1+x3+x5
Intercept	0.333 (0.373)	2.146^*^ (0.976)	0.184 (0.345)	1.858 (0.981)	0.407 (0.387)	1.411 (1.511)
ToM_id	−1.491^*^ (0.592)			−1.201 (0.613)	−1.247^*^ (0.618)	−1.188 (0.619)
FL_FB		−5.058^*^ (2.286)		−3.733 (2.292)		−2.576 (3.758)
DL_FB			−3.543^*^ (1.678)		−2.617 (1.684)	−1.053 (2.774)
p	0.003	0.006	0.009	0.002	0.003	0.007
AIC	50.923	51.991	52.668	49.374	49.721	51.227
BIC	54.301	55.368	56.046	54.440	54.788	57.983

Significance: * indicates p < 0.05.

**Y: ASD diagnosis (0: Neurotypical, 1: ASD)**

**x1: ToM index**

**x2: Proportion of correct first looks (TB condition)**

**x3: Proportion of correct first looks (FB condition)**

**x4: Differential looking time score (TB condition)**

**x5: Differential looking time score (FB condition)**

**Y ~ x1 + x2 + x3 + x4 + x5 (for regression analysis and model selection)**

The models selected in the regression analysis were, in order of the maximum BF value, Y~x1+x3 ([BF=12.741, adj. R2 = 0.225]), Y~x1+x5 ([BF=10.553, adj. R2 = 0.215]) and Y~x1 ([BF=9.215, adj. R2 = 0.172]). The stepwise method of model selection revealed that first, adding x1 (ToM index) resulted in a BIC difference of −4.906. Next, adding x3 (FL_FB i.e., correct first look ratio in the false belief condition) resulted in a BIC difference of −0.0126, indicating that these combinations were the optimal models for the minimum BIC [p=0.00339, adj. R2 = 0.225]. In the correlation matrix of variables (**Figure 5**), the correlations between x2–x5 were greater than 0.5. Since there was a considerable amount of multicollinearity, which can be a variance widening factor, combining pairs of these measures (x2–x5) is not recommended.

The ASD diagnosis was strongly correlated with the ToM index (obtained from the OD task) and the AL measurements (especially the correct first look ratio in the FB condition or DLTS in the FB condition). Stepwise methods indicate that the best model to explain the ASD diagnosis would combine the ToM index and one of the AL measurements rather than using the ToM index alone.

## 4 Discussion

The purpose of the present study was to test the reliability and convergent validity of the implicit FB tasks and their usefulness for ASD diagnosis. We developed a standardized, reliable, and valid implicit ToM measurement. This method is a combination of two typical conventional implicit measures, the AL and OD methods, and is expected to be an improved implicit task that exploits the strengths of both methods. In recent years, there have been many challenges associated with the AL method, especially its fragility and unreliability, due to its low engagement and high exclusion rate. In addition, the results of similar AL methods were inconsistent across tasks, which raised the question of whether there is a (homogeneous) concept of spontaneous ToM that is tapped into by different tasks (15, 21, 31). For the OD method, which was originally developed by Kovacs, Téglás, and Endress et al., some vulnerabilities of the task have also been noted, such as the confounding factor of the attention-grabbing sound stimuli in the movie for reaction time and the inability to ignore psychological refractory periods due to trial repetition (32). To overcome these problems, we inserted the AL method into the OD paradigm without any sound stimulus and used repeated trial averages as AL measurements. These procedures enabled us to obtain robust data by offsetting the inherent fragility of the task. For the purpose of eye movement measurement, the face was fixed, and the viewpoint was set to the agent’s viewpoint (similar to the OD method) to create a more engaging environment. Furthermore, by using a 2-alternative, forced-choice paradigm, in which the reaction time was measured by tipping the left and right levers, the target AOI was placed on the same screen in all conditions, thereby minimizing differences between task conditions. These modifications were made in accordance with the conventional method, and the results generally replicated the results of previous studies in a neurotypical group. The first look ratio was significantly greater for correct AOIs than for incorrect AOIs in the TB condition for both the neurotypical and ASD groups, and although there was no significant difference between the two in the FB condition, there was a trend towards correct > incorrect AOIs in the neurotypical group and vice versa in the ASD group. The DLTS results replicated those in previous studies by Senju (11) and Schneider et al. (29). And Wu et al. (33) also succeeded in replicating positive results with a multi-trial paradigm, which suggests that increasing the number of trials not only effectively reduced the high dropout rate but also decreased error variance. The reaction time (RT) results were significantly different from those of FB (P−A+) in the neurotypical group when TB (P−A−) was used as the baseline, and no such difference was found in the ASD group. In addition, the RTs in the FB (P+A−) condition tended to be longer in the neurotypical group and, conversely, shorter in the ASD group, but this result is consistent with the trend that the greater the agent’s FB attribution, the longer the RTs in the FB (P+A−) condition.

In the present study, multiple measures were obtained in a single paradigm, allowing a within-subject comparison of multiple measures, and the results showed that each measure of the AL and OD methods was robust and independent. The correlation analysis of both methods revealed a significant correlation. In the neurotypical group, correlations were found in both the TB and FB conditions. However, the ASD group showed weak correlation in the FB condition, probably because the distribution of DLTS data was centralized. These correlation results suggest that both tasks exhibit the same ToM ability. Furthermore, we also conclude that the consistency between the results of the AL method as the pre-evaluation measure and the OD method as the post-evaluation measure suggests that there is a time-fixed correlation between the two. In other words, it is possible that an anticipatory gaze at FBs directly affects reaction speed. In the regression analysis, no multicollinearity was found between the measures of the AL and OD methods. Although no measurements were correlated with AQ scores, ASD diagnosis was correlated with any of the measurements. Furthermore, the results of the regression analysis and stepwise model selection revealed that the model equation for an ASD diagnosis that combined two or more measures significantly increased the degree of fit compared with that of each measure alone. In other words, not only the measure of the OD method (ToM index) but also those of the AL method were found to contribute effectively to the ASD diagnosis. These results suggest that the measures of the AL and OD methods are complementary to each other which derive from the same spontaneous ToM ability. It should be noted that the AL and OD methods have never been directly compared and validated in the context of ASD diagnosis before.

There are certain observations regarding the functional brain similarities between the AL and OD methods. fMRI studies have shown that reaction times involving FBs are associated with the right temporal-parietal junction (rTPJ) (34–40). The AL method has also been shown to activate regions around the TPJ in implicit as well as explicit tasks (41). In general, the TPJ is spatiotemporally located between the visual cortex and the mPFC in the network involved in mentalizing (42), and the dmPFC is activated when explicit FB attributions are made in mentalizing tasks (43–45). Inherently, the TPJ and frontal cortex are also closely involved in the acquisition of ToM abilities, and the ToM network, especially between the rTPJ and the prefrontal cortex, is considered as an important neural basis for the emergence of a full-fledged ToM around age 4 (46). Even if the counterargument is that implicit FB tasks are not tasks that require higher-order cognitive functions, the involvement of the TPJ is at least obvious from previous imaging studies, and in the immediate context, implicit FB attribution abilities play a central role in communication. Thus, innate functional differences in TPJ emerge in social situations that require immediate spontaneous communication, which is important for ASD diagnosis.

The prevalence of developmental disorders has increased in recent years (47, 48), especially as more cases of developmental disorders are first diagnosed in adulthood, with a large population being missed below the threshold (49). There is still a lack of measurements that can serve as diagnostic markers (50). Although many meta-analyses of implicit FB tasks have resulted in a widespread negative view of these tasks owing to their high variance across tasks, it is desirable that the results of this direct comparison between tasks provide a reevaluation of the usefulness of FB tasks. Although Nijhof et al. were unable to demonstrate consistency between ASD diagnosis and the behavioral data (39, 40), we successfully demonstrated more robust behavioral data on ASD diagnosis or not, and we expect this paradigm to be extended to a variety of subjects and to be neurologically supported in the future.

As a limitation, the sample size of the present study was determined based on statistical calculations (26) but was not yet large enough to provide normative data split by factors such as IQ, the AQ, sex, and age. Currently, a larger-scale examination of the issue is underway, led by Schuwerk et al. (registered in Child Development in 2021). In addition, our scenarios were monotonous, discrete stimuli of short duration, which does not allow for an expanded interpretation of how individuals would respond in a scenario with more contextual information. Another problem with the task structure is that the intertrial interval is as short as 2 seconds, so the effect of the psychological refractory period cannot be ignored, and the variability caused by lever toppling (the levers have play) cannot be accounted for. The characteristics of Schneider et al.’s experiment were that they dared to avoid paying attention to the moving images so as not to let the subjects explore the intention of the story (29), but our experiment, on the contrary, required the subjects’ engagement.

Our task was more in line with real-life communication styles, as it required immediate and spontaneous responses within a short time constraint. Spontaneous and immediate mentalizing ability is a core component of communication problems. This study is important not only for the pathophysiology of ASD but also for elucidating the basic mechanisms of social communication skills, and further research on implicit ToM abilities is warranted.

## 5 Conclusion

Inspired by the possibility that implicit FB tasks could help diagnose ASD (10), we efficiently combined two representative methods of implicit tasks. The results revealed a generally positive replication of previous research and significant correlations with an ASD diagnosis. Importantly, the implicit nature of this experiment resulted in robust results with low exclusion rates with repeated data. This method provided an independent assessment of the general individual characteristics that distinguish ASD from neurotypical individuals. These findings can potentially contribute to the future development of gaze bias or reaction time biomarkers as diagnostic tools for ASD.

## Data Availability

The raw data supporting the conclusions of this article will be made available by the authors, without undue reservation.
