# Supplementary material for: Spontaneous theory of mind in autism: are anticipatory gaze and reaction time biases consistent?
Source: Front Psychiatry. 2024 Dec 9;15:1189777. doi: 10.3389/fpsyt.2024.1189777 (PMC11663880; doi:10.3389/fpsyt.2024.1189777)
Supplement: Supplementary file 1 [file DataSheet1.pdf]

Supplementary Material

Spontaneous Theory of Mind in Autism: Are Anticipatory-Gaze and Reaction-time Biases Consistent?

Keigo Onda[1,2], Rizal Ichwansyah[1,2], Keisuke Kawasaki[2], Jun Egawa[1], Toshiyuki Someya[1]*, Isao Hasegawa[2]*

*** Correspondences:**
Isao Hasegawa
isaohasegawa@med.niigata-u.ac.jp
Toshiyuki Someya
psy@med.niigata-u.ac.jp

d1 False Belief Test

1.1 Anticipatory Looking (AL) (Supplementary Figures. 1–7)

This paradigm examines anticipatory eye-movement data by presenting participants with movies of a FB task. The movies contain an agent and an object, and as the object moves, true or false beliefs about the object's location are formed depending on whether the agent is looking or not. Participants watching the movie thus unconsciously exhibit spontaneous eye movements while watching an "agent tracking an object hidden in one of two locations," and the eye-movement data during this process are measured. Specifically, in the agent's process of FB formation, the agent first witnesses a scene in which an object is hidden on one of two sides. Then, the object moves to the other side while the agent is absent. This creates a false belief in the agent's mind, namely, a mismatch between the agent's assumed location of the object and the actual location. Next, the agent reappears, the windows (AOIs) on both sides light up, and the movie is stopped for a short time. We measure which side the participant looks at first (proportion of first looks) and which side the participant looks at more during the eye-movement measurement period (DLTS). These values are used as measurements to evaluate the extent of implicit FB attribution.

1.2 Object Detection test (OD)

The object detection test is closely related to the violation of expectation (VOE) paradigm or the altercentric bias paradigm. The VOE task might involve participants watching a movie in which an object is moved from its original location (A) to another location (B) while the agent is absent. When the agent returns, if they find the object in A (violating the expectations of participants), the participants are surprised and spend more time gazing at the movie. The altercentric bias task is expected to provide the following findings. That is participants are slower and more error-prone in performing a given task when the perspective of another agent who is present but irrelevant to the task is inconsistent with their own (relative to when it is consistent) (Samson, 2010). The OD makes use of this tendency. In the present study, we will use a similar paradigm, the OD, in which participants will be asked to pull a lever as soon as possible in the direction of the location where the

object reappears at the last scene (c.f., the original method used by Kovacs; button-pressing). The reaction time from the object's reappearance to the participant response will be measured. This task will consist of scenarios that contain unnatural depictions, such as an agent looking in a different location than participants' belief (true or false) or an object reappearing (or not) and will be used to evaluate whether there is a difference in the reaction time between the natural and unnatural scenarios. The presence/absence (+/-) of the object according to the beliefs of the participant (P) and the beliefs of the agent (A) can be varied to generate four conditions: P+A+, P-A-, P+A-, and P-A+. The first two conditions are the TB conditions, and the latter two are the FB conditions. In the P-A- condition, reaction time should be slowed by "surprise" when the object reappears in violation of the participants' expectations. The key is the difference in reaction times in the P-A+ condition, in which the participants know that there is no object, but the agent falsely believes that there is an object (Figs. 8 and 9 of Supplementary Figure 1). Kovács et al. (2010) concluded that participants' recognition of the agent's false beliefs and the resulting sustained spatial attention do not prolong the reaction time when the object reappears in an unexpected location. This method aligns with the spatial attention paradigm developed by Posner (1980).

2 Pilot Data

A hybrid paradigm combining both AL and the OD (see Methods for details) was designed to determine whether neurotypical adults exhibit significant gaze bias and reaction-time bias indicating implicit attribution of false beliefs to agents in movies and whether these two successively acquired measures are consistent within individuals. In each movie, the agent searched for a hidden object, the location of which may be consistent (TB condition) or inconsistent (FB condition) with the agent's knowledge, depending on the movement of the object when the agent was absent or looking away. The first half of our movie was used for the AL paradigm, and the second half was used for the OD. Specifically, subject eye movements during a 2,000-ms period after the reappearance of the agent were analyzed to detect anticipatory looks to the left and right areas of interest (AOIs) where the agent correctly or incorrectly believe the object to be hidden. Gaze bias in the TB condition indicated that the subject anticipates others' TB-driven actions, whereas gaze bias in the FB condition indicated that the subject implicitly attributes false beliefs to others. The video was terminated when the subject pulled a lever to the left or right side after the object reappeared at the end of the video. Shorter reaction times to the reappearance of the object in the AOI congruent with the agent's false beliefs would indicate implicit FB attribution by the subject. Since the AL paradigm measures anticipatory eye movements before the reappearance of an object (pre-evaluation) and the OD measures reaction times after the reappearance of the object (post-evaluation), our paradigm had the advantage of directly testing the consistency between these two sequentially-acquired measures in the same individuals. Ten participants aged 20 years or older were included in the neurotypical adult group (3 of whom were female, age 27.4±2.41 years) in the pilot study. The order in which the movies were presented, the direction in which the agent turned to look away, and the position of the object's appearance were counterbalanced. Three of 10 participants were excluded due to failure of familiarization. Following object reappearance, all participants pulled the lever in the correct direction with an accuracy of over 90% (98.8 ± 1.28%). As a supplementary psychological test, the Autism-Spectrum Quotient (AQ) (Baron-Cohen, 2001) was conducted (mean score, 17.0 ± 5.07).

We first divided all data into four time bins to examine the learning effect across trials within subjects (Supplementary Figure 2). A two-way ANOVA indicated that there were no significant main effects of the condition (TB or FB condition) [Bayes factor [BF] = 0.277 for first correct look ratio,

BF = 0.267 for the DLTS], time bin [BF = 0.168, BF = 0.173, respectively] or an interaction between the condition and time bin [BF = 0.311, BF = 0.300, respectively]. Post hoc multiple comparisons via two-tailed tests were not significant [Holm test, BF < 1 for both time-bin combinations]. A two-way ANOVA indicated that there was a significant main effect of condition (P+A+, P-A-, P-A+, P+A-) [BF = 188.837 (BF = 23.541 for P+A+ < P-A-, 22.030 for P-A+ < P-A-, 15.453 for P+A+ < P+A-; BF < 3 in other combinations)]. There was no significant main effect of time bin [BF = 0.191] or a significant interaction between the condition and time bin [BF = 0.058]. These results indicated that there was no learning effect for any variable (the first look ratio, DLTS, or reaction time) or condition, consistent with the negative findings of Schneider et al. (2013) regarding learning effects. Therefore, the data analyzed in this experiment were summarized as trial averages for each condition. However, to analyze the correlation between the DLTS and reaction time, data for each trial were used.

Regarding the proportion of correct first looks in the pilot experiment (Supplementary Figure 3A), a Bayes factor analysis of the two-way ANOVA (contrast model averaging, validity level = 3) showed that the BF value for the main effect of condition was negligible [BF = 0.392], and the BF value for the main effect of correct/incorrect first looks was very strong [BF = 5701.934]. The BF value for the interaction was negligible [BF = 0.485]. These findings supported a main effect of correct/incorrect first looks, as the mean of correct first looks (0.663) was substantially greater than that of incorrect first looks (0.219). A t test was conducted on the proportion of correct first looks using a chance level of 0.5; no significant difference was found in either condition (TB or FB condition) [t = 1.81, df = 6, p value = 0.12, BF = 1.058, 95% CI: 0.440-0.903 for TB; t = 1.87, df = 6, p value = 0.11, BF = 1.1176, 95% CI: 0.452-0.854 for FB]. Considering the small sample size of the pilot study and the fact that there were no-look trials in which participants did not look at either the left or right AOI, the chance level should be less than 0.5; thus, the null hypothesis should not be tentatively accepted based on this result. A similar BF analysis for the DLTS showed that the BF value for the main effect of condition was not significant [BF = 0.464] (Supplementary Figure 3B). Additionally, no significant difference was found from chance (0, indicating no difference in looking time) in either condition (TB or FB condition), indicating that only a trend was observed [t = 2.34, df = 6, p value = 0.058, BF = 1.786, 95% CI: -0.0139-0.636 for TB; t = 2.45, df = 6, p value = 0.05, BF = 1.992, 95% CI: 0.000467-0.584 for FB]. Third, regarding the reaction time (OD measurement), a BF analysis of the two-way ANOVA was performed for each condition level (P+A+, P-A-, P-A+, P+A-), and the BF value for the main effect of condition provided strong evidence [BF=19.847, error=0.59%]. Multiple comparisons (two-tailed, Holm's method) using paired means for each level revealed that the mean reaction time in the P+A+ condition (643 ms) was significantly smaller than that in the P-A- condition (743 ms), and the mean reaction time in the P-A- condition (743 ms) was significantly larger than that in the P-A+ condition (654 ms) [BF=6.263]. Thus, the P-A- condition was the baseline, and there was not only a difference between the P+A+ condition and the P-A- condition but also a significant difference between the P-A+ condition and the P-A- condition. These results are similar to previous studies by Kovács et al. (2010) In addition to the participants' own beliefs, the agent's beliefs influenced the RTs of the participants to some extent, suggesting that simply seeing the agent automatically made participants compute their beliefs and that the agent's beliefs were represented and sustained similarly to the participants' own beliefs.

Next, to examine the relationship between the AL and OD paradigms, a correlation analysis was conducted using trial-by-trial values of the DLTS and reaction time (Supplementary Figure 4). Reaction time was normalized for each subject. The results showed moderate to strong evidence in support of correlations in the TB (P+A+) condition [r=-0.328, BF=14.221, ρ_95% CI: -0.483 - -0.094], TB (P-A-) condition [r=0.344, BF=5.578, ρ_95% CI: 0.066-0.500], and FB (P-A+) condition

[r=-0.262, BF=3.101, ρ_95% CI:-0.432 - -0.03]. Thus, there was a significant correlation between the two paradigms, indicating that anticipatory gaze, reflecting belief attribution to the agent, strongly affected reaction time. These results are tentative due to the small sample size of the pilot phase; the present study with a larger sample may verify these findings.

In addition, to examine the contribution of each measurement to the AQ score, a regression analysis was conducted with the AQ score as the dependent variable and each measurement as the independent variables (Supplementary Figure 5 and Supplementary Table 1). Then, a BF analysis was conducted for the following interaction models, and model selection was performed.

Y: AQ (Autism-Spectrum Quotient)

x1: ToM index (reaction time difference between the P−A− and P−A+ conditions)

x2: Proportion of correct first looks (FB condition)

x3: Proportion of correct first looks (TB condition)

x4: Differential looking time (TB condition)

x5: Differential looking time (FB condition)

Y ~ x1 + x2 + x3 + x4 + x5 (for regression analysis and model selection)

The AQ score was strongly correlated with the ToM index (obtained from the OD) and weakly correlated with the AL measurements. Ideally, the best model to explain the AQ score would combine the ToM index and an AL measurement rather than the ToM index alone.

References

Baron-Cohen, S., Wheelwright, S., Skinner, R., Martin, J. & Clubley, E. (2001). The autism-spectrum quotient (AQ): evidence from Asperger syndrome/high-functioning autism, males and females, scientists and mathematicians. J. Autism Dev. Disord. 31, 5–17. doi: 10.1023/a:1005653411471.

Kovács, Á.M., Téglás, E., and Endress, A.D. (2010). The social sense: susceptibility to others' beliefs in human infants and adults. Science. 330:1830-1834. doi: 10.1126/science.1190792

Posner, M.I. (1980). Orienting of attention. Q. J. Exp. Psychol. 32:3-25. doi: 10.1080/00335558008248231

Samson, D., Apperly, I. A., Braithwaite, J. J., Andrews, B. J. & Bodley Scott, S. E. (2010). Seeing it their way: evidence for rapid and involuntary computation of what other people see. J. Exp. Psychol. 36, 1255–1266.

Schneider, D., Slaughter, V.P., Bayliss, A.P., and Dux, P.E. (2013). A temporally sustained implicit theory of mind deficit in autism spectrum disorders. Cognition. 129:410-417. doi: 10.1016/j.cognition.2013.08.004

Supplementary Figures and Tables

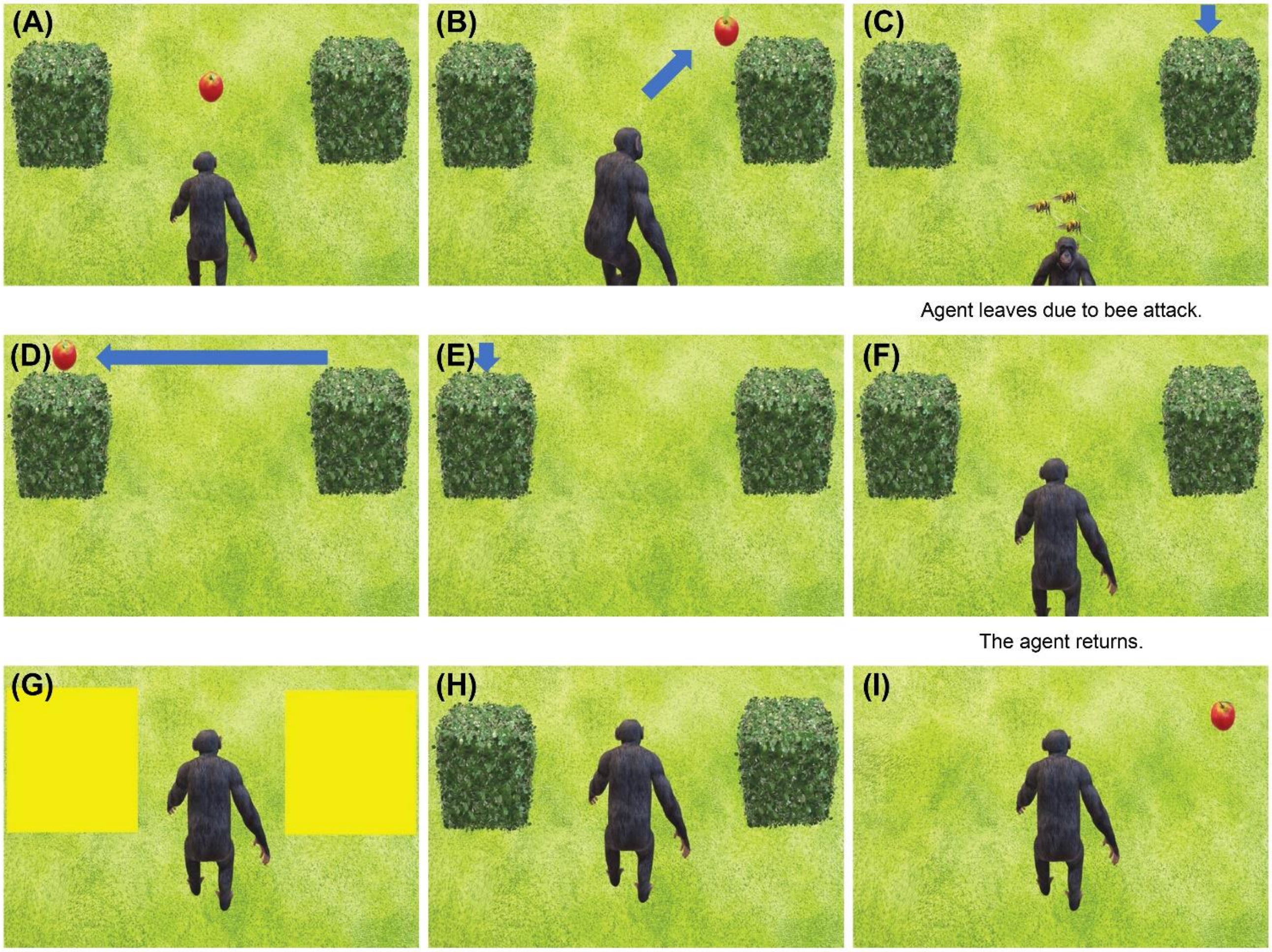

Supplementary Figure 1. Screenshots from selected frames displaying the FB condition (P−A+)

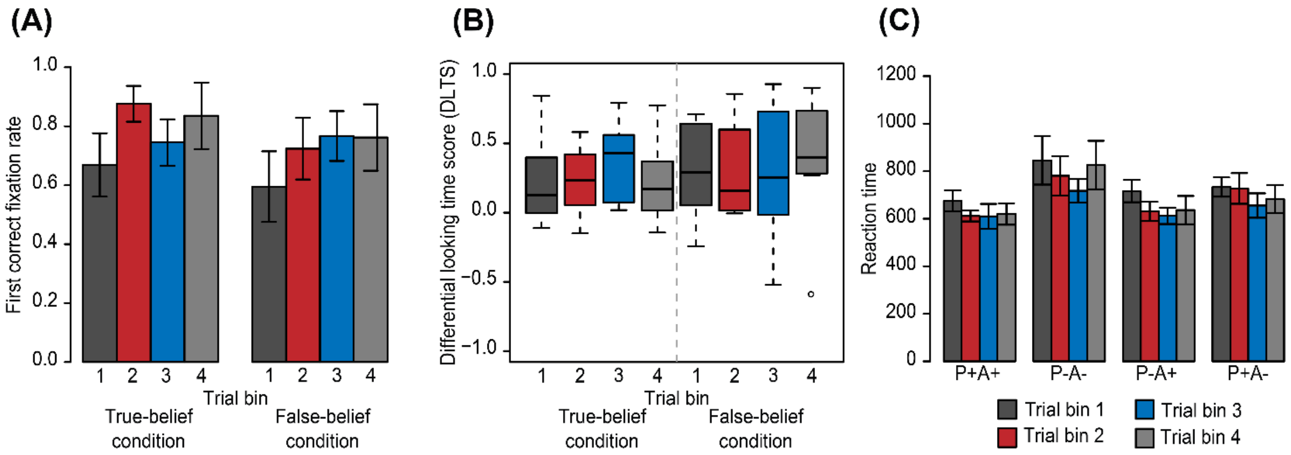

Supplementary Figure 2. Proportion of correct (i.e., congruent with the agent's belief) first looks in the true or false belief conditions in each time bin (**A**). Differential looking time score (DLTS) (**B**). Reaction times in the P+A+, P−A−, P−A+, and P+A− conditions (**C**). Error bars represent the standard error of within-subject effects. N=7.

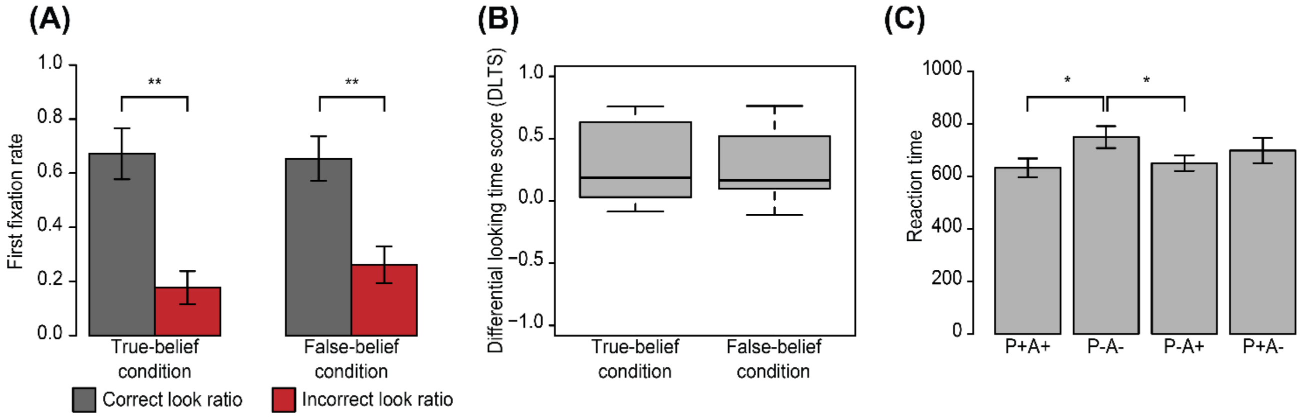

Supplementary Figure 3. Proportion of first looks (correct or incorrect) for each condition (**A**), DLTS (**B**), and reaction times in the P+A+, P−A−, P−A+, and P+A− conditions (**C**). Error bars represent the standard error of within-subject effects. ** indicates a BF > 10, and * indicates a BF > 3. N=7.

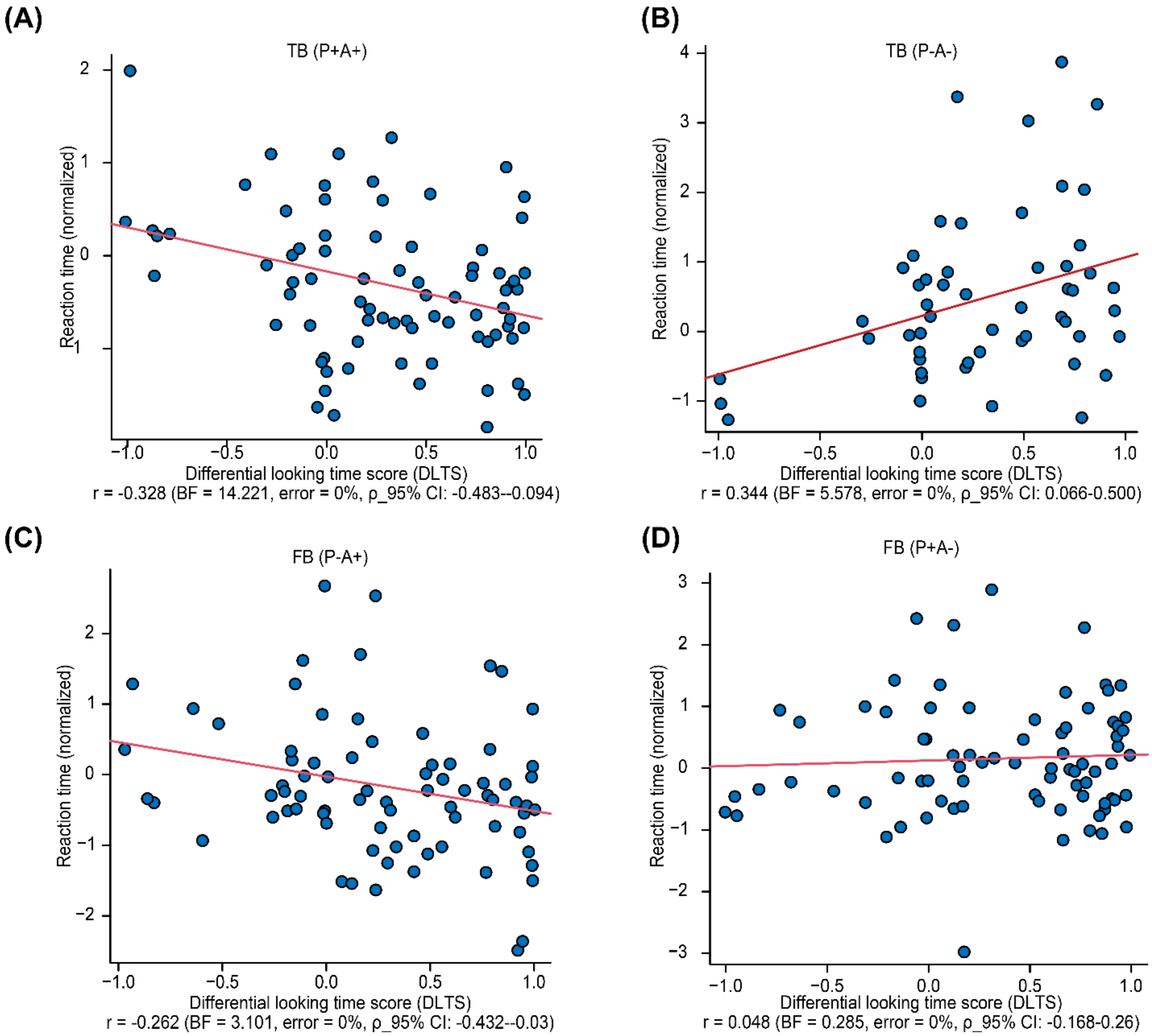

Supplementary Figure 4. Relationship between the DLTS and reaction times in each trial in each condition. N=7.

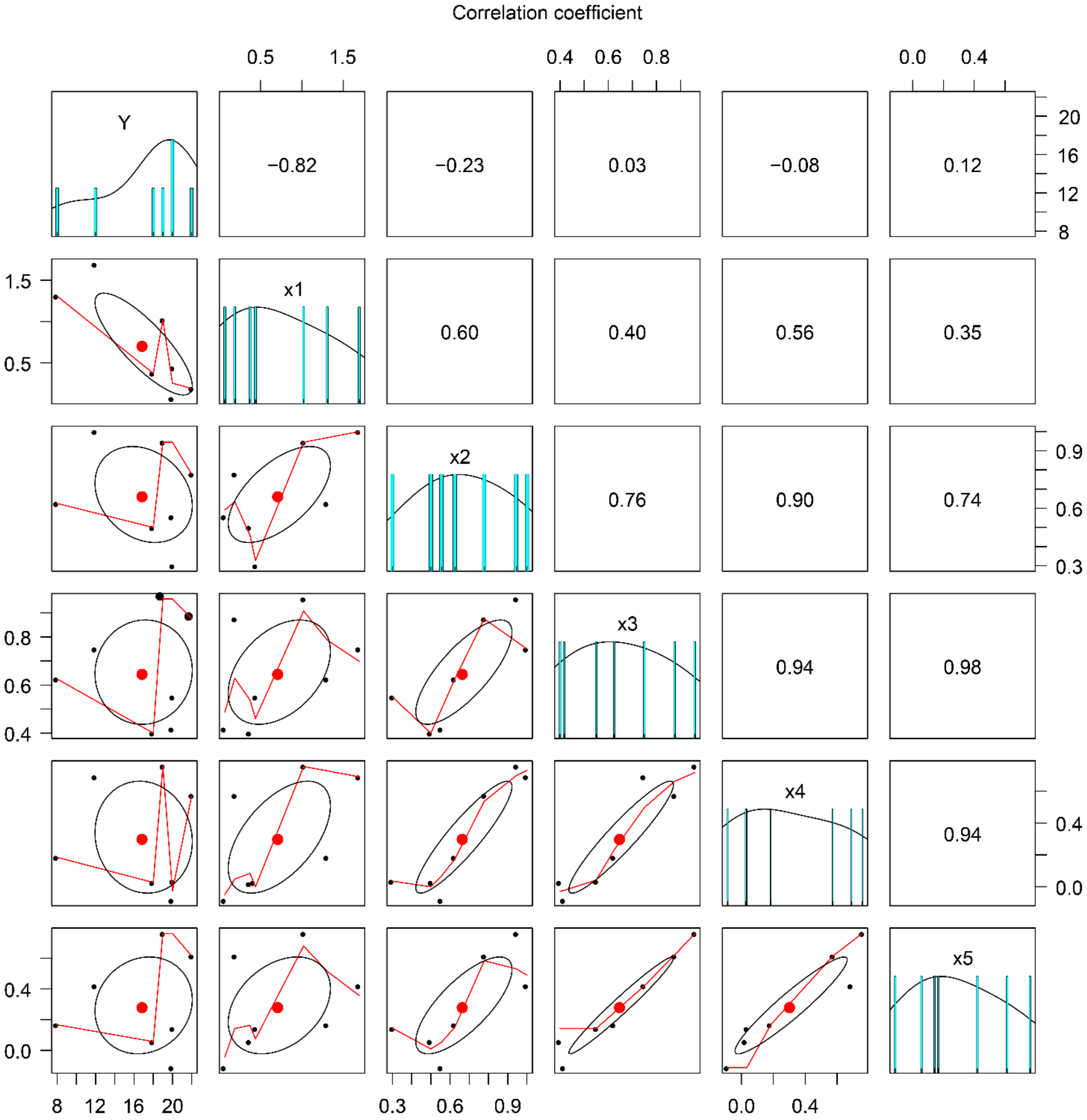

Supplementary Figure 5. Correlation coefficient between the AQ score (on the Y axis) and all measurements (on the X axis). N=7.

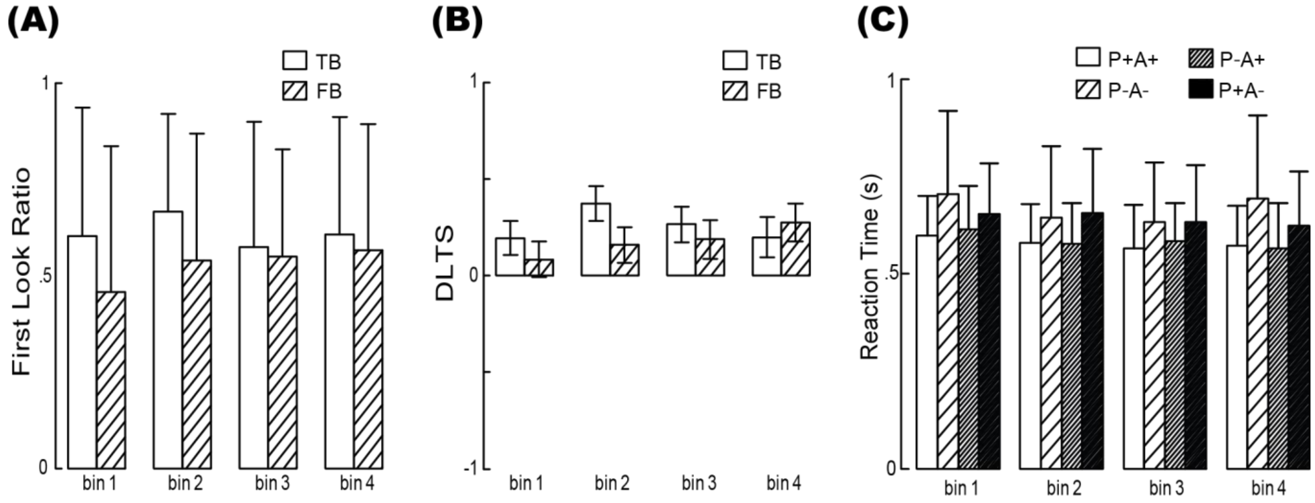

Supplementary Figure 6. Proportion of correct (i.e., congruent with the agent's belief) first looks in the true or false belief conditions in each time bin (**A**). Differential looking time score (DLTS) (**B**). Reaction times in the P+A+, P−A−, P−A+, and P+A− conditions (**C**) in neurotypical group. Error bars represent the standard error of within-subject effects. There were no significant differences between time bins in each condition. N=20.

Supplementary Table 1. Regression results and model selection with the AQ score as the dependent variable and the ToM index (x1) and DLTS in the FB condition (x5) as the independent variables.

	Y~x1	Y~x5	Y~x1+x5
(Intercept)	17.000***	17.000***	17.000***
	(1.207)	(2.083)	(0.893)
x1	-6.714*		-8.035**
	(2.113)		(1.669)
x5		1.900	7.383
		(7.121)	(3.259)
x1 × x5			
R^2	0.669	0.014	0.855
N	7	7	7
BF	2.953	0.553	3.898
AIC	39.766	47.403	35.988

Significance: *** indicates p < 0.001, ** indicates p < 0.01, and * indicates p < 0.05.

Supplementary Table 2. Regression results and model selection with the AQ score (Y) as the dependent variable and the ToM index (x1), FL (x3) and DLTS (x5) in the FB condition as the independent variables**.** N=40. ToM_id, ToM index; DL_FB, DLTS in the FB condition; FL_FB, FL in the FB condition; AIC, Akaike's Information Criterion; BIC, Bayesian information criterion.

	Model (n = 40)					
	Y~x1	Y~x3	Y~x5	Y~x1+x3	Y~x1+x5	Y~x1+x3+x5
Intercept	0.038***	0.030***	0.038***	0.032***	0.038***	0.039***
	(0.002)	(0.005)	(0.002)	(0.006)	(0.002)	(0.009)
ToM_id	0.006			0.005	0.005	0.005
	(0.003)			(0.003)	(0.003)	(0.003)
FL_FB		0.022		0.016		−0.003
		(0.012)		(0.013)		(0.023)
DL_FB			0.019*		0.015	0.017
			(0.009)		(0.010)	(0.017)
p	0.460	0.491	0.424	0.686	0.641	0.828
AIC	298.384	298.858	297.744	298.959	298.011	299.991
BIC	303.450	303.925	302.811	305.714	304.767	308.436

Significance: *** indicates p < 0.001, ** indicates p < 0.01, and * indicates p < 0.05.
